# A Novel Framework for Cardiovascular Disease Detection Using a Hybrid CWT-SIFT Image Representation and a Lightweight Residual Attention Network

**DOI:** 10.3390/diagnostics16010005

**Published:** 2025-12-19

**Authors:** Imane El Boujnouni

**Affiliations:** 1 Laboratory of Information and Communication Technologies, Abdelmalek Essaadi University, Tangier 90000, Morocco; imane.elboujnouni@etu.uae.ac.ma; 2 Laboratory of Innovation in Management and Engineering, Institut Supérieur d’Ingénierie et des Affaires (ISGA), Rabat 10090, Morocco

**Keywords:** electrocardiogram (ECG), cardiovascular disease (CVD), continuous wavelet transform (CWT), Scale-Invariant Feature Transform (SIFT), residual attention network, focal loss

## Abstract

**Background:** The mortality and morbidity rates of cardiovascular disease (CVD) are rising sharply in many developed and developing countries. CVD is a fatal disease that requires early and timely diagnosis to prevent further damage and ultimately save patients’ lives. In recent years, numerous studies have explored the automated identification of different categories of CVDs using various deep learning classifiers. However, they often rely on a substantial amount of data. The lack of representative training samples in real-world scenarios, especially in developing countries, poses a significant challenge that hinders the successful training of accurate predictive models. In this study, we introduce a framework to address this gap. **Methods:** The core novelty of our framework is the combination of Multi-Resolution Wavelet Features with Scale-Invariant Feature Transform (SIFT) keypoint density maps and a lightweight residual attention neural network (ResAttNet). Our hybrid approach transforms one-dimensional ECG signals into a three-channel image representation. Specifically, the CWT is used to extract hidden features in the time-frequency domain to create the first two image channels. Subsequently, the SIFT algorithm is implemented to capture additional significant features to generate the third channel. These three-channel images are then fed to our custom residual attention neural network to enhance classification performance. To tackle the challenge of class imbalance present in our dataset, we employed a hybrid strategy combining the Synthetic Minority Over-sampling Technique (SMOTE) with Edited Nearest Neighbors (ENN) to balance class samples and integrated Focal Loss into the training process to help the model focus on hard-to-classify instances. **Results:** The performance metrics achieved using five-fold cross-validation are 99.60% accuracy, 97.38% precision, 98.53% recall, and 97.37% F1-score. **Conclusions:** The experimental results showed that our proposed method outperforms current state-of-the-art methods. The primary practical implication of this work is that by combining a novel, information-rich feature representation with a lightweight classifier, our framework offers a highly accurate and computationally efficient solution, making it a significant step towards developing accessible and scalable computer-aided screening tools.

## 1. Introduction

Cardiovascular diseases (CVDs) have been listed by the World Health Organization (WHO) as the primary contributor to deaths globally. CVDs account for around 31% of all deaths globally, resulting in millions of deaths annually. Moreover, the financial burden of CVDs is substantial, amounting to billions of dollars globally.

Cardiovascular disease is a multifactorial complex disease influenced by a broad range of physiological and behavioral risk factors. CVDs are associated with potentially modifiable physiological factors, including high blood glucose, high blood pressure, hypertension, high body mass index (BMI), and excessive cholesterol [1]. Among the non-modifiable risk factors are age, genetic predisposition or heredity, and type 1 diabetes [2]. Moreover, genetic risk has been demonstrated to play a significant role in overall cardiovascular risk [3].

Thus, with a view to slowing the current rate of mortality and mitigating the negative impacts of CVDs, early diagnosis and timely treatment are of paramount importance. The most frequent types of cardiovascular diseases include coronary artery disease, myocardial infarction, congestive heart failure, bundle branch block, valvular heart disease, and dilated and hypertrophic cardiomyopathy [4]. These cardiac conditions commonly cause morphological disturbances of the ECG waveforms, leading to irregular heartbeats and abnormalities in heart rhythm.

### Etiologies of CVDs

This section gives a brief description of the etiology of each cardiovascular disease examined in this study.

- Congestive heart failure (CHF) refers to a condition that occurs when the heart is unable to pump blood properly through the body [5], which is generally the result of a range of underlying reasons, including myocardial infarction, valvular heart disease, coronary artery disease, cardiomyopathy, and hypertension.
- Valvular heart disease (VHD) refers to a dysfunction in any of the four valves, namely the tricuspid, pulmonary, aortic, or mitral valve. This results in restricted blood flow or blood leaking back into the ventricles. The defects in these valves are usually due to congenital malformations or acquired from other factors such as aging or infections [6].
- Coronary artery disease (CAD) refers to the blockage of the coronary arteries responsible for delivering oxygen and nutrients to the heart muscle. This obstruction arises from the accumulation of fatty materials such as fat and cholesterol [7].
- Myocardial infarction (MI), or a heart attack, occurs due to a blockage of blood circulation to part of the heart, leading to irreversible damage to the cardiac muscle. This interruption of blood circulation is due to the blockage of a coronary artery by a ruptured atherosclerotic plaque, which is a buildup of cholesterol and other substances in the artery walls [8].
- Cardiomyopathy is a condition that impacts the cardiac muscles and walls. This often results from a thickening of the walls of the heart between the two ventricles, particularly the left ventricle, leading to several problems that affect the heart’s function [9].
- Bundle Branch Block (BBB) is a condition where the normal conduction of electrical impulses in the heart is slowed or interrupted. It is classified into Right Bundle Branch Block (RBBB) and Left Bundle Branch Block (LBBB) [10].
- Myocarditis is an inflammatory condition that affects the myocardium, the middle layer of the heart wall. This inflammation can be triggered by diverse factors, most commonly viral infections such as influenza, coxsackievirus, and parvovirus B19. Other pathogens, including viruses, bacteria, fungi, and parasites, can also lead to myocarditis. Non-infectious causes include autoimmune disorders, exposure to certain toxins or medications, and hypersensitivity reactions. This condition impairs the heart’s ability to pump blood, which can lead to serious complications such as heart failure and arrhythmias [11].
- Dysrhythmia is a disturbance in the normal electrical activity of the heart, which generally manifests as the heart beating too fast, known as tachycardia, or beating too slow, referred to as bradycardia. The etiology of dysrhythmias can be associated with various underlying heart diseases, including coronary artery disease, cardiomyopathy, or valvular heart disease. It may also be caused by electrolyte imbalances in the blood, injury to the heart muscle from a myocardial infarction, or as a consequence of cardiac surgery [12].

**Figure 1:**
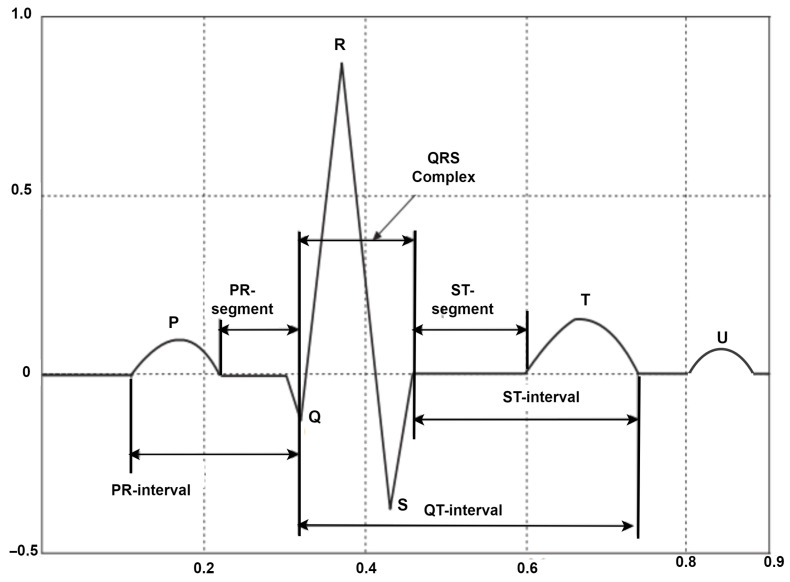
Visual representation of the normal ECG wave structure.

The electrocardiogram is a widely used non-invasive tool utilized to monitor the electrical impulses as they appear on the body surface. Particularly, as the heart beats over time, the ECG provides relevant insights into the time-varying bioelectric potential produced by the electrical activity. This technique generates an ECG waveform characterized by six distinct points, namely P, Q, R, S, T, and U waves, with each wave corresponding to a distinct phase of the cardiac cycle. The normal ECG waveform, known as Normal Sinus Rhythm (NSR), exhibits five points: P, Q, R, S, and T waveforms. A visual representation of the heart rhythm waves and temporal intervals is illustrated in Figure 1.

The P wave is the initial wave in the ECG cycle resulting from the electrical activity during atrial depolarization, which causes the atria to contract and propel blood into the ventricles.

The PR interval is recorded in the ECG cycle from the onset of the P wave to the onset of the QRS complex and reflects the conduction time for the electrical impulse to propagate from the SA node through the atria, the atrioventricular (AV) node, and the bundle of His before spreading to the ventricles. The normal PR interval range can vary between 120 and 200 ms.

The QRS complex is composed of the Q, R, and S waves and indicates ventricular depolarization, leading to their contraction and pumping blood into the circulatory system. The QRS complex duration ranges from 0.08 to 0.10 s.

The ST segment begins at the end of the S wave and ends at the beginning of the T wave and has a duration ranging from 0.08 to 0.12 ms. This segment appears as an isoelectric line and reflects the period between ventricular depolarization and repolarization.

The T wave is a small wave that appears after the QRS complex and represents the repolarization of the ventricles. The normal duration of the T wave ranges between 120 ms and 160 ms.

The QT interval starts from the onset of the QRS complex to the end of the T wave and reflects the duration of both ventricular depolarization and subsequent repolarization.

The U wave is a minor waveform that appears after the T wave and may not be readily visible in a significant proportion of ECGs, typically around 50% to 75%, because it is obscured by the T wave and the imminent new P wave. It is hypothesized that the U wave is caused by depolarization of the interventricular septum.

The electrocardiogram (ECG) is considered the primary diagnostic tool employed by physicians and doctors to detect possible abnormal conditions in ECG heartbeats. Notwithstanding, the process of assessing cardiac electrical activity and performing a careful analysis with the naked eye requires substantial human resources and is a time-consuming process. Besides this, the manual process is highly prone to errors and may result in ECG misinterpretation, particularly when dealing with substantial amounts of data. To overcome these challenges, researchers have explored and investigated the use of computer-aided cardiac decision tools, which can potentially enhance the diagnostic performance of cardiac disease by detecting subtle variations in the parameters of ECG waveforms. Therefore, as a screening tool, these systems can assist physicians and cardiologists in making more informed decisions and enhancing overall classification accuracy.

In the past decades, numerous research studies have been carried out to investigate the automatic detection of cardiac diseases using computer-aided tools. Most existing research reported in the literature emphasizes binary classification that distinguishes between two classes of CVDs, including MI, CHF, CAD, VHD, and BBB, along with normal classes; only a limited number of studies have investigated the automated categorization of multiple classes.

Deep learning algorithms can automatically learn, process, and pick up discriminative features from ECG data. There are various forms of DL algorithms that have been explored in the classification of CVDs; the most common forms include the convolutional neural network (CNN), long short-term memory network, recurrent neural network, and hybrid CNN-LSTM. Convolutional Neural Networks (CNNs) represent a widely used form of deep learning classifier and have been utilized to implement diagnostic computer-aided systems for identifying different categories of cardiovascular diseases. Although these CNN-based methods have reported some promising results in the diagnosis of different heart diseases, they heavily depend on the availability of a large volume of training data to obtain higher success rates [13], which is a time-consuming and very computationally intensive process. Furthermore, in healthcare analysis and especially in the categorization of heart diseases, insufficient representative training data in real-world scenarios poses a significant and serious obstacle to building accurate predictive models. Although substantial amounts of ECG data are generated every day in hospitals, much of this data is unannotated, and the manual annotation by specialist physicians is considered a laborious and time-consuming process. Furthermore, the requirement of significant computational resources by deep, complex CNNs makes them unsuitable for deployment in developing countries. To implement them in mobile and wearable devices, it is essential to reduce network parameters and optimize the memory footprint. Hence, this highlights the necessity for a strategy that ensures efficient modeling and accurate prediction using lightweight neural networks.

Motivated by these challenges, in this study, we develop an automated computer-aided system for accurately identifying ten cardiovascular classes: myocardial infarction (MI), valvular heart disease (VHD), coronary artery disease (CAD), congestive heart failure (CHF), dysrhythmia (DY), myocarditis (MYO), hypertrophic cardiomyopathy (HCM), dilated cardiomyopathy (DCM), bundle branch block (BBB), and a normal (N) class. To achieve this, we propose a novel approach that utilizes a hybrid representation of Multi-Resolution Wavelet Features combined with Scale-Invariant Feature Transform (SIFT) keypoint density maps to capture discriminative features from the ECG signals. To process these features, we developed an advanced deep Convolutional Neural Network (CNN) architecture, the Residual Attention Network (Ranet).

In summary, the primary contributions presented in this study can be summarized as follows:

- To the best of our knowledge, this study is the first to introduce an automated system for classifying nine distinct classes of cardiovascular diseases in addition to a normal class.
- To the best of our knowledge, this study is the first to utilize a combination of Multi-Resolution Wavelet Features with Scale-Invariant Feature Transform (SIFT) keypoint density maps to capture the most discriminative spectral features, thereby enhancing classification performance.
- We developed a lightweight residual attention neural network (ResAttNet) to effectively enhance diagnostic performance.
- To alleviate the issue of class imbalance and mitigate the risk of biased learning, we adopt a multi-faceted strategy. Specifically, we employed a hybrid technique combining the Synthetic Minority Over-sampling Technique (SMOTE) with Edited Nearest Neighbors (ENN) to enhance class distribution and improve data quality, while Focal Loss is adopted to assign higher weights to minority classes and reduce weights for majority classes.

The rest of this study is arranged as follows: Section 2 presents an updated literature review of the state-of-the-art studies focusing particularly on feature extraction and selection techniques, alongside the application of machine learning and deep learning models. Section 3 provides an in-depth analysis of the different stages involved in the full procedure; we thoroughly describe the employed experimental settings and protocols. A discussion of experimental results and the comparison of our results with the state-of-the-art studies are examined in Section 4. The conclusions of the work are summarized in Section 5.

## 2. Related Works

A broad range of feature extraction methods has been proposed to capture valuable information from ECG signals. These features can be derived from several domains, including the time domain, frequency domain, or other domains.

The authors in [14] explored the Autoregressive Burg algorithm in time-frequency analysis to extract feature vectors. The Higher-Order Statistics and Spectra method was employed by researchers in [15] to extract higher-order statistical bispectrum and cumulant features from ECG heartbeats. A novel two-band optimal biorthogonal filter bank was proposed in [16]. The Hilbert–Huang transform was implemented in [17] to identify features from short-term ECG signals. The authors in [18] exploited several feature extraction methods, including Shannon entropy, binary particle swarm optimization, and Shearlet and Contourlet transformations. In [19], eight handcrafted features were selected from eight different domains, including time, amplitude, frequency, energy, higher-order statistics, and entropy. The authors in [20] employed temporal and beat-to-beat morphological features from ECG signals. In [21], the dual-tree complex transform was implemented to extract useful features. A feature fusion-based technique was explored in [22] to extract the features of 12-lead ECG signals. The authors in [23] treated multi-lead ECG signals as 2D matrices to be input into the CNN model for multi-scale feature extraction.

Other researchers have investigated the use of deep neural networks to perform automated feature extraction tasks. Deep networks employed their inherent self-learning capabilities to process and learn nonlinear features from ECG signals without requiring explicit manual feature extraction. Specifically, both feature extraction and classification are combined and performed within DNN-based classifiers. However, it is worth noting that this automated feature extraction requires considerable computational resources and a large dataset during the training phase. Several authors have focused on applying deep networks for the automated extraction of important features, such as in [18,19,24,25,26,27] where the authors opted for CNN models, while others employed hybrid CNN models [28,29,30], or LSTM models [31].

In the past decades, a substantial number of statistical and machine learning algorithms have been implemented for the classification of CVDs [15,16,18,20,32,33,34,35], including naïve Bayes, decision trees, random forests, support vector machines, linear regression, and K-nearest neighbor.

The authors in [18] developed a computer system for the automated detection of four classes (N, CHF, MI, CAD) using ECG heartbeats. Specifically, k-nearest neighbor and decision tree classifiers were used in the classification stage. The best accuracy achieved was 99.55% using the KNN classifier trained with the contourlet coefficient. An AI-based method was proposed in [32]; the authors employed a naïve Bayes algorithm to estimate the one-year risk in patients with DCM disease. Support vector machines and random forest classifiers were used in [20] to identify patients with HCM disease from 10-second ECG signals. They attained a sensitivity of 85% and a specificity of 90%.

The authors in [16] employed KNN classifiers for the classification of patients with MI disease, resulting in an accuracy of 99.62%. The authors in [15] classified CHF using various machine learning algorithms, namely k-nearest neighbor, decision tree, support vector machine, random forest, and artificial neural network. The highest accuracy of 100% was obtained by a random forest algorithm.

These ML algorithms employed handcrafted feature extraction and selection methods from the ECG signals, which depend on domain knowledge and require careful consideration. Moreover, they are often associated with a time-consuming and laborious process. Therefore, in recent years, several researchers have increasingly oriented themselves toward deep learning methodologies to tackle the aforementioned issues of ML techniques.

The convolutional neural network (CNN) is a special form of deep learning algorithm that has been widely utilized for ECG-based CVD identification, where both the feature extraction and classification stages are integrated and automatically performed together. In particular, multiple convolutional layers are cascaded successively to extract the underlying features of ECG signals. However, they often rely on a significant amount of ECG data in the training process. The authors in [15,18] developed a deep convolutional neural network of 11 layers for the detection of coronary artery disease and congestive heart failure, using 2-second and 5-second ECG segments.

In [23], the authors designed a model called multi-lead-CNN to identify anterior myocardial infarction using heartbeat segments, resulting in an accuracy of 96%, specificity of 97.37%, and sensitivity of 95.4%. In [27], the authors proposed a classification system to diagnose HCM patients using a CNN classifier; they obtained a sensitivity of 93% and a specificity of 87%. Some authors implemented hybrid deep models using CNNs. For example, in [36], the authors developed an automatic multi-channel algorithm by combining LSTM and 16-layer CNN classifiers to form a hybrid solution for MI diagnosis using ECG heartbeats. They obtained an accuracy of 95.54%, sensitivity of 98.2%, and specificity of 86.5%. The authors in [37] combined a 16-layer Deep Convolutional Neural Network with a Recurrent Neural Network to build a hybrid model for myocardial infarction detection based on 12-lead ECG signals, achieving an accuracy of 99.9%. In [28], the authors integrated a 16-layer CNN and LSTM classifiers for the categorization of three distinct classes of CAD, CHF, and MI using 2-second ECG segments. Their method yielded an accuracy of 98.51%, sensitivity of 99.30%, and specificity of 97.89%. A deep 1D convolutional neural network was proposed in [26] for the automated detection of five classes using various ECG segment intervals. The best performance results were achieved using three-second segment durations with an accuracy of 99.84%, sensitivity of 99.52%, and specificity of 99.95%. The authors in [25] implemented an automated system based on CNN and unique Gabor CNN models for the automatic identification of four ECG signal classes: normal, MI, CHF, and CAD, resulting in an accuracy of 98.74%, sensitivity of 98.74%, and specificity of 99.46%. In [38], the authors developed an automated computer-aided system for identifying eight categories of cardiovascular diseases using lead II ECG signals; they explored using the CWT to convert the heartbeats into scalograms. These images are then further processed through the DWT, resulting in wavelet coefficient images serving as input to a custom convolutional capsule network. They achieved an accuracy of 98.6%, precision of 99.1%, sensitivity of 99.47%, and F1 score of 99.4%.

## 3. Materials and Methods

**Figure 2:**
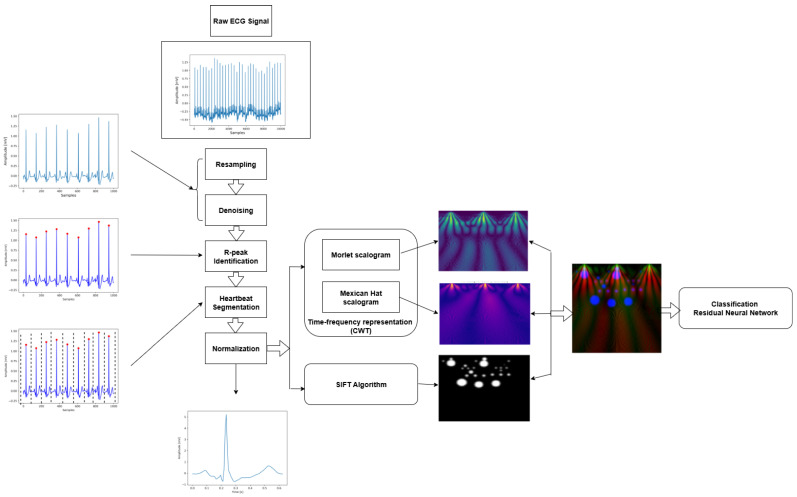
Flowchart illustrating the proposed method, where an ECG signal is converted into combined scalogram and SIFT features for classification with a Residual Neural Network.

This section provides an in-depth analysis of the proposed approach for the automated classification of CVDs. As depicted in Figure 2, the approach is divided into four primary stages: data loading, data preprocessing, followed by feature extraction, and classification. The preprocessing stage is divided into two parts: ECG signal resampling and denoising. The feature extraction stage includes three parts, namely segment extraction, normalization, and the hybrid fusion that combines Multi-Resolution Wavelet Features (MRS) with SIFT keypoint density maps. In the following sections, we discuss each part within these stages in detail.

### 3.1. Dataset Description

Raw ECG signals were retrieved from three different open-source databases in the Physionet bank [39] to validate the performance of the proposed method. The databases are the Physikalisch-Technische Bundesanstalt (PTB), St. Petersburg Institute of Cardiological Technics (INCART), and the Beth Israel Deaconess Medical Center Congestive Heart Failure (BIDMC) databases. Table 1 provides an overview of the ECG data retrieved from each respective database, including information such as the type of ECG signal, number of subjects, sampling frequency, and number of leads.

- The PTB Diagnostic ECG database, obtained from the Physikalisch-Technische Bundesanstalt (Germany), is widely recognized as one of the largest publicly available databases, comprising 12-lead ECG recordings with various profile information, such as gender, age, and health information. The database includes 549 ECG recordings obtained from 290 participants, with 209 men and an average age of 57.2 years. Within the database, 52 subjects are classified as healthy, whereas 148 subjects present various cardiac conditions. Each subject contributes one to five ECG records, with each record containing 12-lead ECG signals. Only lead II was employed in this study. Eight disease types, including myocardial infarction, dysrhythmia, valvular heart disease, bundle branch block, dilated cardiomyopathy, myocarditis, hypertrophic cardiomyopathy, and the normal (N) class, were extracted from the PTB diagnostic database.
- The St. Petersburg Institute of Cardiological Technics 12-lead Arrhythmia (INCART) database contains 75 annotated recordings derived from 32 Holter records and obtained from 32 distinct individuals (17 males; 15 females; aged 18–80). The database was similarly recorded using a 12-lead ECG configuration, wherein 73 ECG recordings have a duration of 30 min and a sampling frequency of 275 Hz. Coronary Artery Disease patients were derived from this database.
- The BIDMC Congestive Heart Failure database consists of 15 subjects with severe congestive heart failure, classified under New York Heart Association (NYHA) functional classes III and IV. Among the participants, there are eleven male patients ranging in age from 22 to 71 years and four female patients ranging in age from 54 to 63 years. The recordings have a duration of approximately 20 h and contain two-channel ECG recordings with a sampling rate of 250 Hz. Congestive heart failure patients were extracted from this database.

### 3.2. Data Preprocessing

#### 3.2.1. Resampling

Since the three databases investigated in this study have different frequencies, signal resampling is highly required in disease classification to ensure uniformity and standardization across all the investigated databases. Accordingly, we initiate the procedure by resampling all the data to a uniform frequency of 128 Hz. This rate is sufficient to preserve all critical diagnostic features, as the most clinically significant information in an ECG signal, including QRS morphology, is concentrated well below the 64 Hz limit imposed by the Nyquist theorem. This creates a consistent dataset that can be accurately used for the classification of CVDs.

#### 3.2.2. Noise Removal

**Figure 3:**
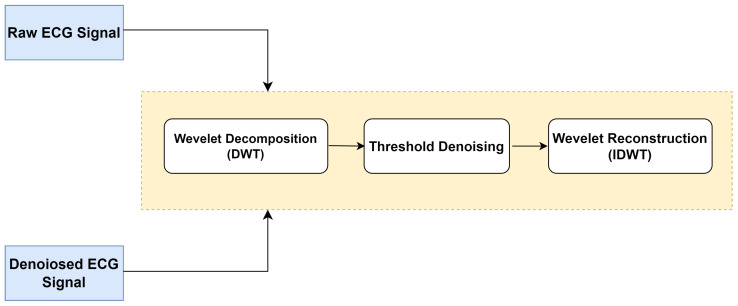
Schematic of the wavelet denoising procedure.

ECG signal acquisition is highly prone to corruption from various sources, which can substantially affect its performance quality. The sources include high-frequency noise, which arises from power line interference, and low-frequency noise, which is the result of body movement, respiration, and electromyography (EMG) [40]. In the context of biometric analysis, effective ECG filtering is crucial to preserving the relevant characteristics of the heartbeat and avoiding misclassification. In this study, a two-step filtering process was applied: first, a Butterworth bandpass filter was used to remove baseline wander and high-frequency disturbances. Second, wavelet-based denoising was employed for further refinement of the signal. This wavelet-based denoising approach combined two techniques, namely the wavelet decomposition–reconstruction process along with the soft thresholding technique. The procedure involved three stages, as depicted in Figure 3.

In the first stage, we perform a wavelet decomposition to efficiently eliminate the baseline drift noise in the raw ECG signal. This noise corresponds to a low-frequency signal ranging from 0.5 to 0.6 Hz. Specifically, in the decomposition process, we employed the Daubechies (db6) wavelet function [41] owing to its waveform similarity with the normal ECG signals. By applying this decomposition, we extract the DWT coefficients for each level, consisting of a set of one approximation coefficient (C5) and five detail coefficients (D1 to D5).

The second stage involves threshold processing of the wavelet coefficients obtained by Multi-Resolution Wavelet Decomposition. Specifically, the soft threshold method is used to mitigate the high-frequency components reflected by the power line interference. The soft threshold method is a nonlinear function used to process the wavelet coefficients of the signal subspace. Coefficients exceeding a predefined threshold are modified using a step function Sgn based on the variation between the coefficient and the threshold value thr, while the coefficients in the noise subspace are set to zero if they are below the predefined threshold value.

The calculation formula for the wavelet coefficient with the soft threshold technique is presented as follows:

(1) $$\delta_{m,n}^{s}\left(x\right)=\left\{\begin{array}{ccc} 0 & \; & \left|x\right|⩽thr\\ Sgn\left(x\right)\left(\left|x\right|-thr\right) & \; & \left|x\right|>thr \end{array}\right.$$

**Figure 4:**
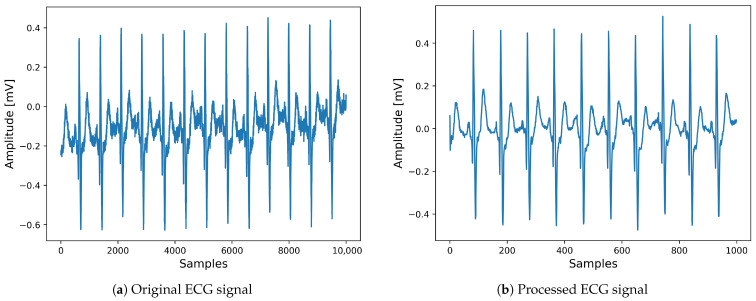
Comparison of (**a**) original ECG signal and (**b**) processed ECG signal.

In the last stage, we reconstruct the denoised ECG signal by performing the inverse discrete wavelet transform (IDWT) on the threshold-processed DWT coefficients. Figure 4 illustrates both the noisy ECG signal and its corresponding filtered version resulting from the adoption of the discrete wavelet transform technique.

#### 3.2.3. Heartbeat Segmentation

**Figure 5:**
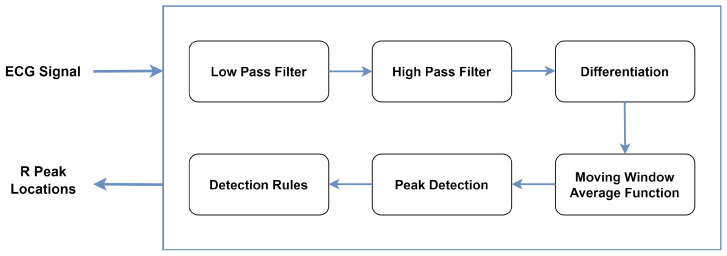
Block diagram of Hamilton algorithm.

An essential step involves the accurate location of R peak points. Various algorithms have been developed to perform this task, but in this study, we employed the open-source Hamilton segmenter algorithm to accurately annotate the corresponding peak points within the signal. We selected this algorithm due to its computational simplicity as well as its availability in the BioSPPy library. It consists of several stages as depicted in Figure 5. Each stage contributes to the effective localization of the R peaks, allowing further analysis of the ECG signal.

**Figure 6:**
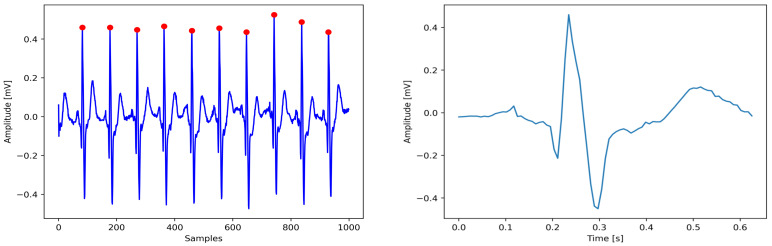
R-peak detection using the Hamilton method, with detected peaks shown as red dots (**left**) and a typical extracted heartbeat (**right**).

**Figure 7:**
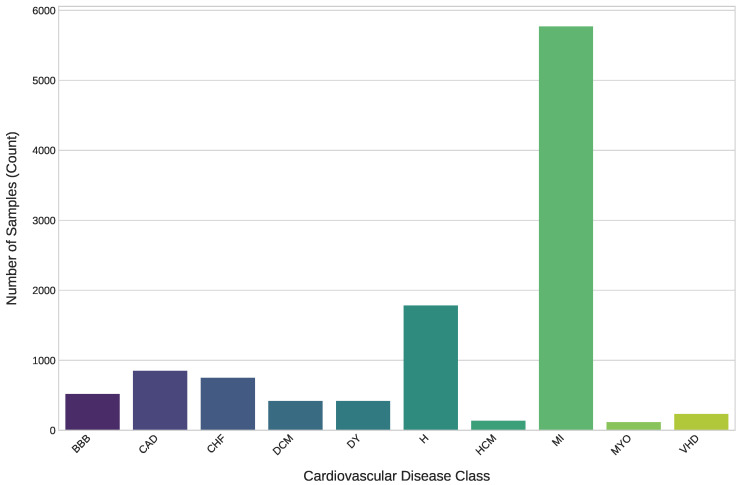
Class distribution of ECG heartbeat samples.

Afterwards, a window of size 640 ms was centered around the R-peak point locations. Specifically, we extracted a window starting 240 ms before the R-peak and extending 400 ms after the R-peak. The locations of R peaks using the Hamilton method and the visualization of an extracted ECG heartbeat are illustrated in Figure 6. A total of 10,990 heartbeat segments were employed in this research. Figure 7 illustrates the imbalanced distribution of classes within the dataset.

#### 3.2.4. Normalization

**Figure 8:**
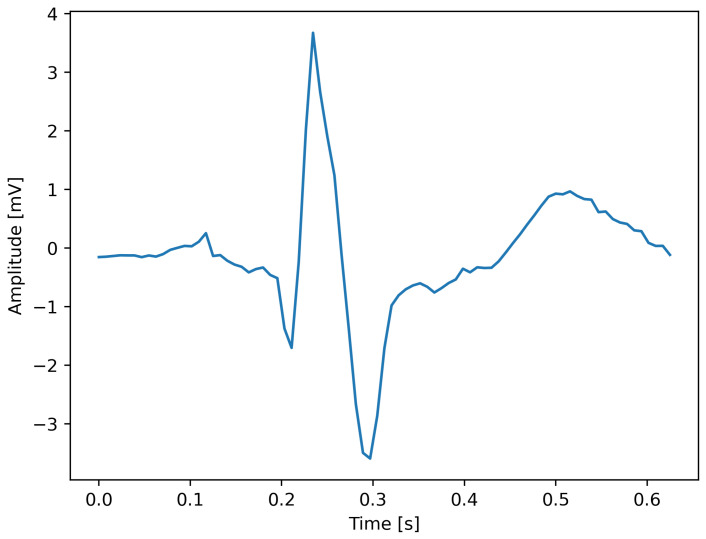
Normalized ECG heartbeat.

The ranges of ECG segment features can vary, and it is highly necessary to mitigate this variability when diagnosing heart diseases to ensure accurate performance. Therefore, the z-score normalization technique is implemented to standardize each ECG segment. This method was chosen over other techniques, such as min–max scaling, due to its robustness to outliers. Physiological signals like ECGs are often subject to noise and artifacts that can create extreme peak values, and z-score normalization is less sensitive to these anomalies. This procedure aims to bring all the segment features into a uniform scale, with a mean of zero and a standard deviation of one. As a result, the offset effect is effectively eliminated, and the values of the segment amplitudes are standardized through squashing the original segment values into a narrower range. A visualization of a normalized ECG heartbeat is illustrated in Figure 8. The z-score normalization technique can be expressed as

(2) $$Z=\frac{x-\mu }{\sigma }$$

The equation involves the normalization of *x*, where the mean value is denoted as μ, and the standard deviation for that category is denoted as σ.

### 3.3. Feature Extraction

The feature extraction stage is a critical step that significantly affects the performance of the classification process. In this study, we propose a novel feature extraction process that combines the continuous wavelet transform (CWT) for time-frequency domain analysis with the Scale-Invariant Feature Transform (SIFT) for effective and robust keypoint detection. The hybrid approach generates a three-channel image representation of each ECG segment.

#### 3.3.1. Continuous Wavelet Transform

To effectively analyze ECG signals, which consist of non-stationary data with various time-varying frequency components, we first employ The Continuous Wavelet Transform, which is an effective and robust technique commonly used in signal analysis because it can provide an overcomplete representation of a signal by continuously varying both the scale and translation parameters. Specifically, in the CWT, the signal is decomposed into a sequence of wavelets, each with a different scale. This technique pioneered the concept of scale as an alternative to frequency analysis, allowing for signal analysis in a time-scale plane.

The time-scale plane employed in the CWT is analogous to the time-frequency plane utilized in the short-time Fourier transform, where each scale corresponds to a specific frequency range in the time-frequency domain. Mathematically, the CWT can be expressed as the convolution of the input signal *x*(*t*) with the analyzing wavelet ψ(t) as given in the following equation:

(3) $$CWT_{s,u}\left(t\right)=\frac{1}{\sqrt{u}}\int_{-\infty }^{+\infty }x\left(t\right)\psi \left(\frac{t-s}{u}\right)dt$$

The CWT produces a time-scale representation of the signal x(t) by computing the convolution at multiple scales, denoted by *s*, and multiple positions, denoted by *u*. The parameter $\frac{1}{\sqrt{u}}$ represents the energy-normalized factor used to normalize wavelets at each scale. Adjusting these parameters can affect the temporal and spectral resolution of the analysis. Specifically, when the scaling parameter is smaller than 1, the wavelet is compressed in the time domain, resulting in higher spectral resolution. Conversely, when the scaling parameter is greater than 1, the wavelet is dilated in time, which leads to high temporal resolution. The graphical representation of the translation and dilation transformations employed in the continuous wavelet transform is referred to as the scalogram, which displays the squared modulus of the CWT wavelet coefficients in a time-scale-based representation as described in the following equation:

(4) $$\Delta t\Delta f\geq \frac{1}{4\pi }$$

and Δf is proportional to the center frequency *f*, which results in the equation as follows:

(5) $$\frac{\Delta f}{f}=C$$

where *C* is a constant. Therefore, CWT can provide high temporal resolution and low spectral resolution in high-frequency domains and high spectral resolution and low temporal resolution in low-frequency domains [42].

**Figure 9:**
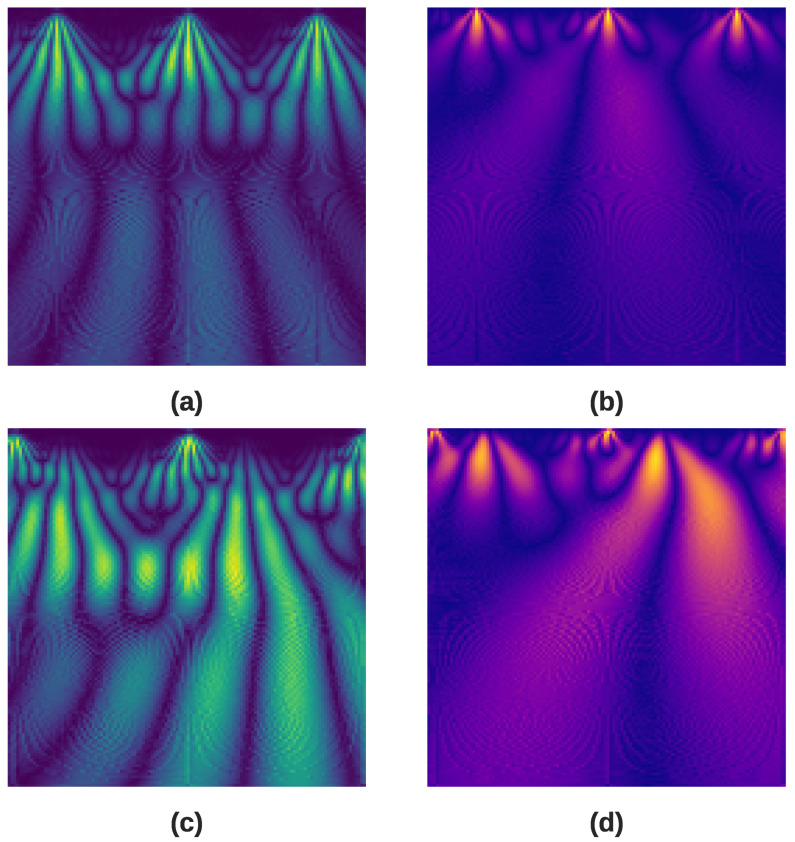
Comparison of time-frequency representations corresponding to two disease classes: (**a**) Morlet scalogram of a myocardial infarction heartbeat; (**b**) Mexican hat scalogram of a myocardial infarction heartbeat; (**c**) Morlet scalogram of a normal heartbeat; (**d**) Mexican hat scalogram of a normal heartbeat.

The scalogram provides a representation of the frequencies present at different times in the signal, with varying colors and intensities indicating the amplitude of frequency components, wherein darker shades of blue represent lower amplitudes, while brighter shades of yellow indicate higher amplitude coefficients. By analyzing the signals across different scales and frequencies using the CWT, hidden features in the time-frequency domain can be revealed, allowing for a more comprehensive ECG analysis. In our implementation, we generate two separate scalograms to create the first two channels of our input image. The first scalogram is derived from the Morlet wavelet, which is good at detecting oscillatory features, while the second scalogram is derived from the Mexican Hat wavelet, which excels at identifying the morphological characteristics of the QRS complex in ECG signals. Figure 9 displays the CWT scalogram images for myocardial infarction and normal heartbeat segments. The columns depict the time-frequency analysis generated using the Morlet and Mexican Hat wavelets, respectively.

#### 3.3.2. Scale-Invariant Feature Transform (SIFT) Algorithm

To further extract significant information in the feature extraction process, the third channel is derived from the Scale-Invariant Feature Transform technique.

**Figure 10:**
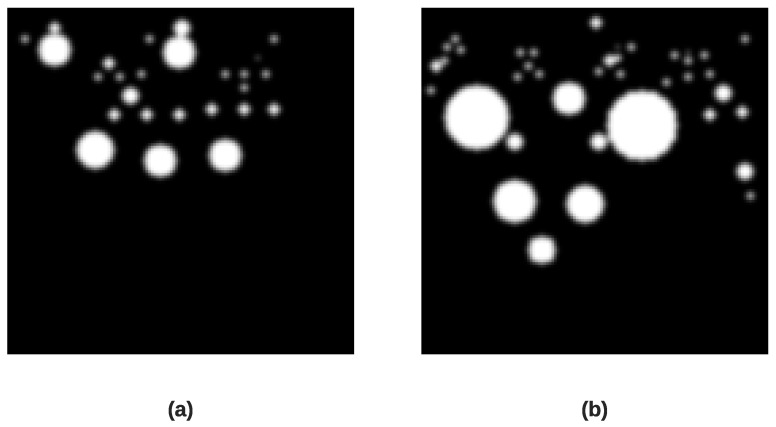
Visual comparison of SIFT-derived feature maps for a myocardial infarction heartbeat (**a**) and a normal heartbeat (**b**). The bright white areas represent regions with a high density of detected keypoints, indicating significant local features in the ECG signal morphology.

**Figure 11:**
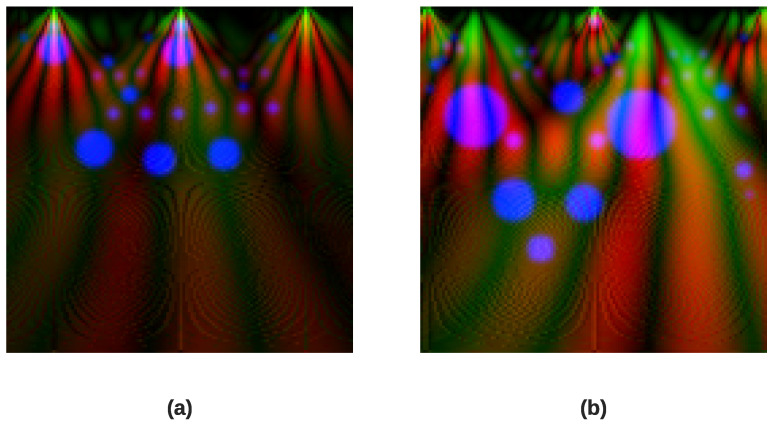
Comparison of the final three-channel feature images for a myocardial infarction heartbeat (**a**) and a normal heartbeat (**b**). The red channel corresponds to the Morlet scalogram, the green channel to the Mexican Hat scalogram, and the blue channel to the SIFT keypoint density map.

The SIFT technique was originally developed for object recognition in computer vision. SIFT is exceptionally robust at identifying distinctive keypoints that are invariant to scale, rotation, and illumination changes. Instead of using SIFT for traditional image matching, we adapt it to create a spatial ’attention map’ that accentuates the most salient regions in the time-frequency representation of ECG heartbeats. The SIFT algorithm proposed in this work involves the following stages:

1. Scale-Space Extrema Detection
  This initial stage detects candidate keypoints by searching for local extrema across multiple scales of the image. The process is achieved through a convolution between the input image, which in our study represents the scalogram image created using the Morlet wavelet, and Gaussian filters at different scales, as expressed in the following equations:
  (6) L(a,b,σ)=G(a,b,σ)×I(a,b)
  (7) $$G\left(a,b,\sigma \right)=\frac{e^{-\frac{\left(a^{2}+b^{2}\right)}{2\sigma^{2}}}}{2\pi \sigma^{2}}$$
  L(a,b,σ) is the scale-space image created by convolving the Gaussian function G(a,b,σ) with the image function I(a,b), which forms a scale-space representation that is prominent for identifying pertinent features across multiple scales. The Gaussian function represents a key component in the convolution process. It consists of a two-dimensional distribution across spatial coordinates *a* and *b*, with its spread controlled by the standard deviation sigma. The Gaussian function’s ability to smooth and blur is a vital step for detecting features across varying scales.
  Keypoints are then effectively identified by searching for local maxima and minima in the Difference of Gaussians (DoG) function, which pinpoints significant image parts, enabling robust detection of keypoints at different scales. The DoG function is created by subtracting the blurred image at a larger scale, kσ, from the blurred image at a smaller scale σ, as expressed in the following equation:
  (8) D(a,b,σ)=L(a,b,kσ)×L(a,b,σ)
  where L(a,b,kσ) represents the convolution interaction between the original image I(a,b) with the Gaussian blur G(a,b,kσ) at scale kσ.
2. Keypoint Localization After identifying potential keypoints at the extrema of the DoG scale-space in the previous stage, they are refined for precision. This refinement is achieved by applying a Taylor expansion D(a), a mathematical technique that helps reject weak and unstable points with low contrast by analyzing the function’s value *D* and its derivatives $\frac{\partial D^{T}}{\partial a}$ and $\frac{\partial^{2}D}{\partial a^{2}}$. This step is fundamental for creating robust and effective scale-invariant descriptors for feature extraction. This is given as follows:
  (9) $$D\left(a\right)=D+\left\{\left(\frac{\partial D^{T}}{\partial a}\right)\times a\right\}+\left\{\frac{a^{T}}{2}\times \left(\frac{\partial^{2}D}{\partial a^{2}}\right)\times a\right\}$$
3. Orientation Assignment
  To ensure rotation invariance, each keypoint is assigned a specific orientation according to the most dominant directions of local image gradients. A histogram of these gradients is created from the pixels surrounding the keypoint. The most dominant peaks in this gradient histogram are assigned as the main orientation of the keypoint. All subsequent operations are performed on image data standardized relative to the scale, location, and orientation of the keypoint, ensuring the final descriptor is invariant to these transformations.
4. SIFT-based Attention Map Generation
  To guide our CNN model to focus on the most relevant features of the scalogram images, we created a SIFT-based attention map. Our approach uses the locations and scales of keypoints in a novel way to create a third channel for our input image, called the SIFT attention map. Specifically, we create this map by plotting a filled circle on a blank 5 × 128 pixel map for each identified keypoint, where the location of the circle is determined by the coordinates of the keypoint and its radius is directly proportional to its detected scale. The resulting map is then smoothed with a 3 × 3 Gaussian filter to reduce noise and create a more continuous representation of keypoint density before being normalized. This new input channel effectively pinpoints the most information-rich regions in the time-frequency representation, creating a data-driven attention mechanism for the CNN.
  Figure 10 displays the resulting SIFT attention maps for a myocardial infarction heartbeat and a normal heartbeat. Finally, Figure 11 depicts the resulted three-channel feature images for myocardial infarction and normal heartbeat.

The specific steps of our CWT-SIFT feature fusion methodology are formally outlined in Algorithm 1.
**Algorithm 1** CWT-SIFT Feature Fusion Process.  1:**Input:** 1D ECG segment, ECGSegment.  2:**Output:** three-channel feature image, FinalImage.  3:**function** GenerateFeatures(ECGSegment)    ▹ Step 1: Generate CWT Feature Channels  4:    Channel1 ← CWT(ECGSegment, wavelet = ’mexh’)  5:    Channel2 ← CWT(ECGSegment, wavelet = ’morl’)                 ▹ Step 2: Generate SIFT Density Map Channel  6:    SIFTBaseImage ← CWT(ECGSegment, wavelet = ’morl’)  7:    GrayscaleImage ← NormalizeToGrayscale(SIFTBaseImage)  8:    Keypoints ← SIFT.detect(GrayscaleImage)  9:    DensityMap ← CreateEmptyMap(height = 5, width = 128)10:    **for** each keypoint ’kp’ in Keypoints **do**11:        (x, y) ← GetScaledCoordinates(kp, target_height = 5, target_width = 128)12:        DensityMap[y, x] ← DensityMap[y, x] + 1       ▹ Populate histogram13:    **end for**14:    BlurredMap ← GaussianBlur(DensityMap, kernel_size = 3 × 3)15:    Channel3 ← Normalize(BlurredMap)                        ▹ Step 3: Combine Channels16:    FinalImage ← Concatenate(Channel1, Channel2, Channel3)17:    **return** FinalImage18:**end function**

### 3.4. Classification

#### 3.4.1. Residual Network

In 2015, the ResNet architecture was proposed by He et al. [42] and has demonstrated remarkable robustness in image classification tasks. Unlike traditional CNNs, ResNet solves the performance degradation problem in deep networks through its unique residual learning.

**Figure 12:**
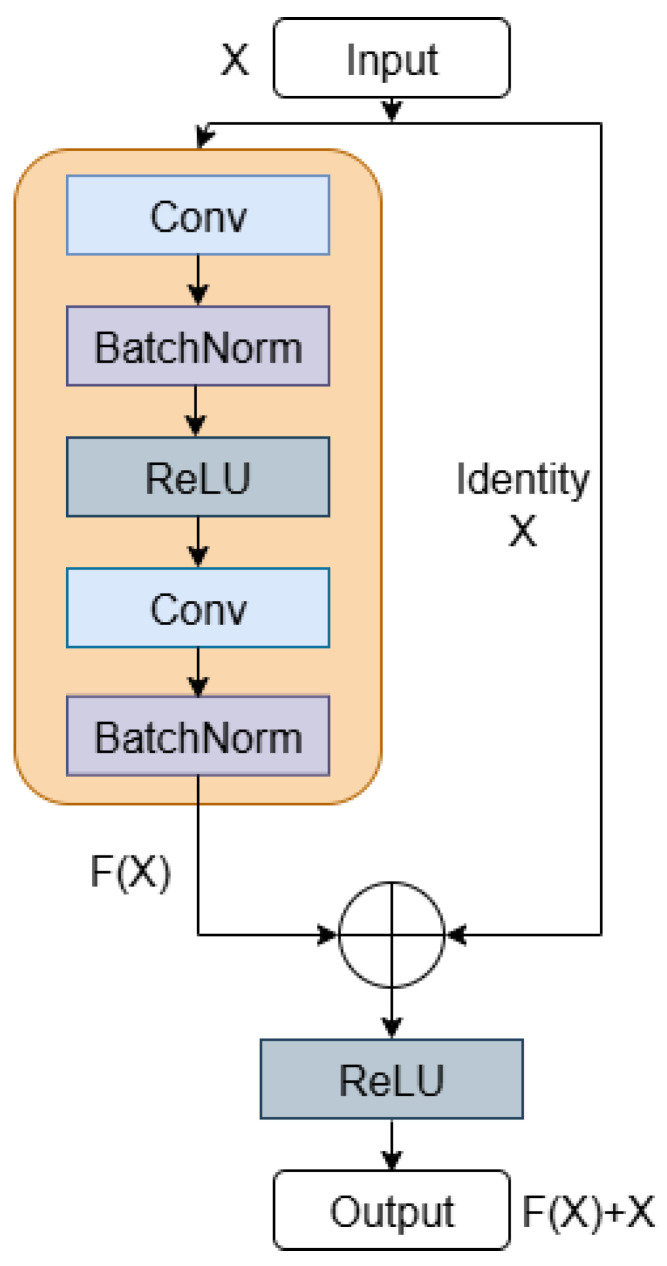
The basic structure of the residual module.

Figure 12 illustrates the basic structure of the residual block in ResNet. The module incorporates two parallel paths, the main path and the residual or shortcut path. The main path is responsible for learning the mapping of the input data, while the shortcut path learns the difference between the input *X* and the desired mapping F(X). These residuals are added back to the original input to produce the final output H(X). This mechanism enables the network to focus on learning the residual information directly, thereby effectively alleviating the vanishing gradient and degradation problems. The operation is given in the following equation:

(10) H(X)=ReLU(F(X)+X)

#### 3.4.2. Proposed Lightweight Residual Attention Network (Light-ResAttNet)

Motivated by the success of ResNet architectures and the need for efficient models that accurately enhance diagnostic performance, we propose a novel customized lightweight architecture, named Light-ResAttNet, specifically tailored for cardiovascular disease classification. The proposed architecture is designed to improve classification performance while maintaining low computational cost, enabling its deployment in real-world scenarios.

**Figure 13:**
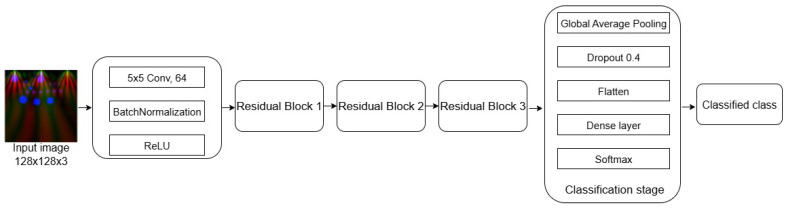
Architecture of the proposed ResNet model. The input is a three-channel image created from Morlet, Mexican Hat, and SIFT feature maps, which is then processed through three residual blocks for classification.

Inspired by the ResNet family, the proposed architecture incorporates several modifications to better handle the characteristics of CVD images. As depicted in Figure 13, the model consists of an initial lightweight convolutional stem, followed by a sequence of pre-activation residual blocks, an embedded spatial attention module, and a classification stage.

**Figure 14:**
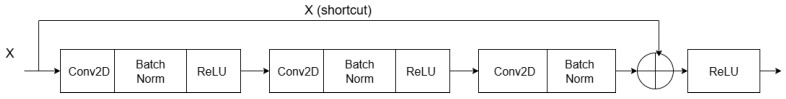
Residual block for the proposed ResNet architecture.

The initial input layer contains a single 5 × 5 convolution with a stride of 2 instead of the standard 7 × 7 kernel used in ResNet18. These smaller filters help in extracting more localized features important for medical image analysis. The residual blocks, as illustrated in Figure 14, adopt a pre-activation structure, where batch normalization and the nonlinear ReLU function precede each convolutional operation. Each block contains two 3 × 3 convolutional layers and an identity shortcut connection. The blocks are organized into three stages with increasing filter dimensions of 64, 128, and 256. To further enhance the ability of the model to focus on the most discriminative spatial regions, we selectively incorporate a spatial attention module within the third residual stage of the network.

Following the final residual stage, the classification head is composed of a Global Average Pooling layer to reduce the spatial dimensions, a Dropout layer to reduce overfitting, and a fully connected SoftMax layer to generate class probabilities across the CVD classes.

The detailed, layer-by-layer architecture of our proposed Light-ResAttNet model is presented in Table 2.

### 3.5. Experimental Setup and Evaluation Metrics

#### 3.5.1. Evaluation Parameters

Model evaluation is a crucial part of determining the efficiency and reliability of the classification results. In this work, four commonly used statistical measures, along with the graphical technique of the ROC curve, were adopted to qualitatively assess the performance results, including accuracy, sensitivity, precision, and F1-score. The definition of these criteria is given below:

Accuracy: The accuracy evaluation metric measures the ability of a classifier to correctly differentiate between classes and is expressed as follows:

(11) $$Accuracy=\frac{TP+TN}{TP+TN+FP+FN}$$

Sensitivity: Sensitivity, recall, or true positive rate (TPR) measures the effectiveness of the model to correctly detect true positive instances. It is defined in the following equation:

(12) $$Sensitivity=\frac{TP}{TP+FN}$$

Precision: Precision, also known as positive predictive value (PPV), indicates the ratio of observation samples from the true positive classes that were correctly classified as positive. It is computed using the following equation:

(13) $$Precision=\frac{TP}{TP+FP}$$

F1-score: The F1-score indicates the mathematical harmonic average of the sensitivity and precision. Specifically, the F1-score measures the total number of correctly identified samples. It is calculated using the following equation:

(14) $$F1-score=\frac{2\times TP}{2\times TP+FP+FN}=2\times \frac{Precision\times Sensitivity}{Precision+Sensitivity}$$

These relevant parameters for evaluating the classification performance are computed based on the values in the confusion matrix, which consists of four elements, namely true positives (TP), false positives (FP), false negatives (FN), and true negatives (TN). Specifically, TP represents the number of positive samples that the classifier correctly identified as positive, while TN denotes the number of negative samples that were accurately classified as negative. FP refers to the number of negative samples that were misclassified as positive, and FN represents the number of positive samples that the model incorrectly labeled as negative.

#### 3.5.2. Experimental Setup

In this study, the five-fold cross-validation technique was employed to assess the reliability, robustness, and performance validation of the designed model.

The experimental process was carried out using the Google Colab notebook, a software platform provided by Google, using a TESLA K80 GPU with a runtime configuration of 12 GB. The proposed model was implemented using the Python language 3.14.0 alongside Keras 3.0.5 with the backend framework TensorFlow 2.16.1.

To monitor the accuracy of the training and validation sets during the learning process, the early stopping mechanism was implemented. Specifically, early stopping occurs if the validation accuracy fails to improve after 10 successive epochs. The model was trained for about 100 epochs using a batch size of 32. We implemented the optimizer Adaptive Moment Estimation (Adam), which can address the issue of local minima. We set the learning rate to 0.001.

To further improve learning under class imbalance ratios, the training pipeline incorporated two complementary strategies: the focal loss function and a hybrid sampling strategy.

Specifically, focal loss [43] reshapes the cross-entropy (CE) loss function to assign higher weights to the difficult-to-classify minority class samples. Accordingly, focal loss improves the classification accuracy of minority class samples and improves overall model performance.

Next, we implemented a hybrid strategy [44] that integrates the Synthetic Minority Over-sampling Technique (SMOTE) alongside Edited Nearest Neighbors (ENN). SMOTE is initially applied to synthetically generate new samples from the minority class, thereby balancing the distribution of classes. Meanwhile, ENN is employed to prune the dataset by removing noisy or misclassified samples, especially within the minority class, thereby improving class quality and separability.

The application of this hybrid strategy resulted in a fully balanced and cleaned training set. Table 3 provides a detailed breakdown of the sample distribution for each class before and after the application of the SMOTE-ENN procedure.

The hyperparameter configurations applied to each component of our experimental framework are detailed in Table 4.

#### 3.5.3. Computational Cost

To assess the practical feasibility of the proposed framework for real-world clinical applications, we evaluated its computational cost. Our Light-ResAttNet architecture was purposefully designed to be lightweight, with around 1.2 million trainable parameters. This is substantially lower than larger and widely used networks such as ResNet-18 with around 11.7 million parameters and ResNet-50 with 25.6 million parameters. The model’s efficiency is also evident in its computational complexity, requiring only 0.09 GFLOPs (Giga Floating Point Operations) per inference, compared with 1.8 GFLOPs for ResNet-18 and 4.1 GFLOPs for ResNet-50.

Furthermore, we measured the model’s inference speed using a Google Colab T4 GPU. The average time required to process a single ECG sample and generate a prediction was 8.85 milliseconds. This combination of high-speed performance and low parameter count suggests that the model is well-suited for deployment in near real-time cardiac monitoring systems, where both speed and accuracy are of paramount importance.

## 4. Results and Discussion

### 4.1. Ablation Experiments

In this section, we perform a sequence of ablation experiments to validate the effectiveness of our proposed feature extraction approach. The aim of these studies is to investigate the individual and combined impact of the different feature extraction approaches used to construct our three-channel image representation.

Accordingly, we designed three baseline experiments from three different aspects. In the first three experiments, the model was trained and tested using only a grayscale single-channel image. The final experiment was conducted to compare these baselines against our final proposed hybrid method that fuses all three channels.

The experimental setups are as follows:

1. CWT with Morlet Wavelet: The model is trained and tested using the time-frequency representation generated by the CWT with the Morlet wavelet.
2. CWT with Mexican Hat Wavelet: The model is trained and tested using the time-frequency representation generated by the CWT with the Mexican Hat wavelet.
3. SIFT Density Map: The model is trained and tested using the grayscale image that represents the SIFT keypoint density map.
4. Proposed Hybrid Method: The model is trained and tested on the final three-channel image, obtained by combining the two CWT channels with the SIFT density map.

The performance of our lightweight residual attention network was evaluated for each of the four configurations using the five-fold cross-validation technique. The overall performance of the results is summarized in Table 5, illustrating the contribution of each study.

A comparison between these results reveals several key insights, indicating that the model that was trained using the three-channel image exhibits a significantly superior and enhanced predictive performance compared with the models trained using single-channel configurations, particularly for minority classes. Specifically, the model trained with the three-channel representation achieves distinguished performance, including an overall accuracy of 99.60% (95% confidence interval: 98.54–99.64%), a sensitivity of 98.53%, a precision of 97.38%, and an F1-score of 97.37%. In contrast, the model trained using the scalograms generated by the CWT with the Morlet wavelet attains an overall accuracy of 96.5%, a sensitivity of 95.6%, a precision of 96.2%, and an F1-score of 96.5%. The model trained with the scalograms produced by the CWT with the Mexican wavelet yields an overall accuracy of 96.3%, a sensitivity of 95.5%, a precision of 95.2%, and an F1-score of 96.5%. Hence, based on the results, the three-channel representation is selected and used as input for the proposed model.

Table 6 presents the detailed performance metrics for each class, providing the accuracy, precision, recall (sensitivity), and F1-score corresponding to each of the classes assessed by the proposed model.

**Figure 15:**
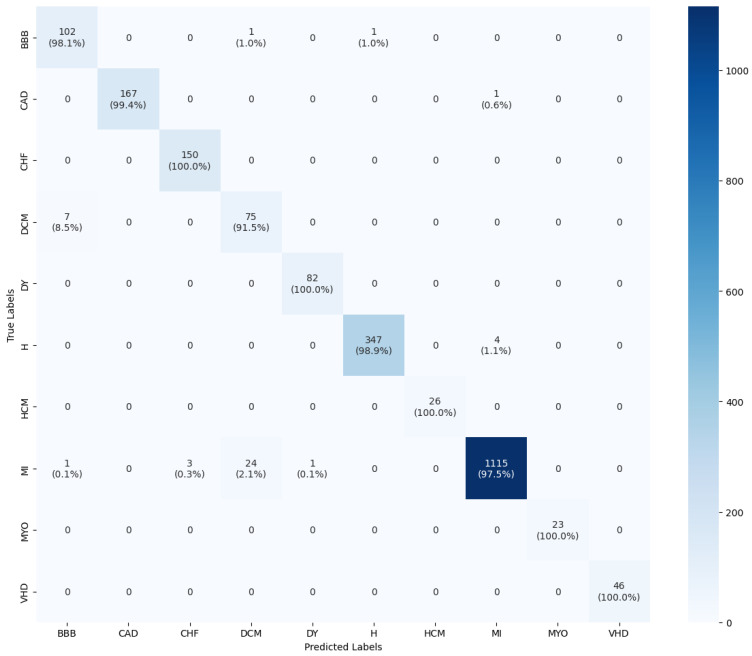
Confusion matrix for the designed model.

Moreover, we evaluate the testing performance of our designed model by analyzing the confusion matrix illustrated in Figure 15. The confusion matrix is a valuable tool used to evaluate a model’s performance, providing a comprehensive summary of the classification counts for true and false predictions within each class. The y-axis represents the actual (true) class labels, while the x-axis represents the predicted class labels. Each square in the matrix shows the number and percentage of predictions for a specific class. The darkly shaded diagonal elements indicate correct classifications, while off-diagonal elements indicate instances of misclassification. Upon examining Figure 15, it can be observed that the model shows only a few misclassifications, notably for the DCM, MI, and BBB classes. Specifically, 8.5% of DCM heartbeats were misclassified as BBB, and 2.1% of MI heartbeats were inaccurately classified as DCM. However, the designed network achieves perfect classifications for the CHF, DY, HCM, MYO, and VHD classes. The 100% correct classification rate for HCM and MYO is particularly significant, as these were the smallest minority classes in the original dataset, which confirms the effectiveness of our SMOTE-ENN balancing strategy.

**Figure 16:**
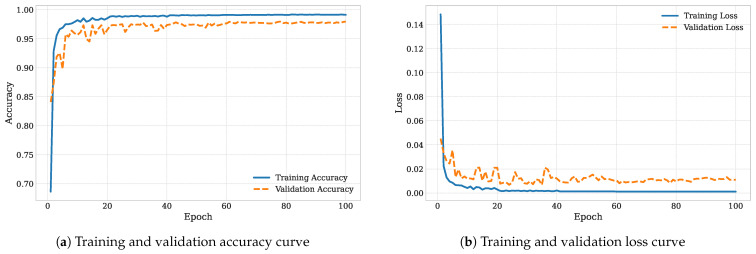
Learning curves of the proposed model showing (**a**) accuracy and (**b**) loss for both training and validation.

To further confirm the stability of the training process, we plotted the training and validation accuracy and loss curves, as shown in Figure 16.

Both curves exhibit smooth convergence, with validation metrics closely following the training ones, indicating that the network generalized well across folds. This observation is consistent with the high per-class performance metrics reported in Table 6 and the confusion matrix analysis.

It is worthwhile to highlight that the designed model yielded a high average accuracy of 99.60% and has demonstrated a good average sensitivity of 98.53%. The developed model aims to reliably detect patients suffering from cardiovascular conditions, which is reflected in this high sensitivity. Furthermore, the model achieves a 98.96% recall for normal (H) heartbeats, proving its ability to accurately distinguish between healthy individuals and patients with diseases. From a clinical perspective, correctly identifying patients with diseases is of high importance, as misclassifying them as healthy may lead to serious cardiovascular complications and potentially delay essential treatment.

In addition to analyzing the confusion matrix, we computed the class-wise counts of true positives (TP), false positives (FP), false negatives (FN), and true negatives (TN) to further understand the model’s behavior. The most significant number of false positives occurred in the DCM class (25 FP), mostly from misclassified BBB and MI instances. Similarly, MI recorded the highest false negatives (29 FN), reflecting some overlap with the DCM, CHF, and DY classes. This specific confusion between MI and DCM is clinically plausible, as both conditions can lead to significant morphological changes in the QRS complex and T-wave abnormalities. The model appears to be capturing these overlapping features, which aligns with clinical realities and highlights the need for future work to incorporate additional data to differentiate between such etiologically distinct but morphologically similar conditions.

We also computed the standard deviation across all classes for FP and FN to assess variability. The standard deviation for false positives was 7.35, and for false negatives, 8.52. These relatively low values indicate a stable performance across classes, with only DCM and MI showing moderate deviations. Notably, classes such as CHF, DY, HCM, MYO, and VHD had no false positives or false negatives, underscoring the classifier’s high precision and recall for these categories.

True negatives for each class were also high, further confirming that the classifier is not over-predicting any single class. Overall, the low FN and FP deviations and high TN counts reflect a consistent and reliable classification performance across all 10 cardiovascular disease categories.

**Figure 17:**
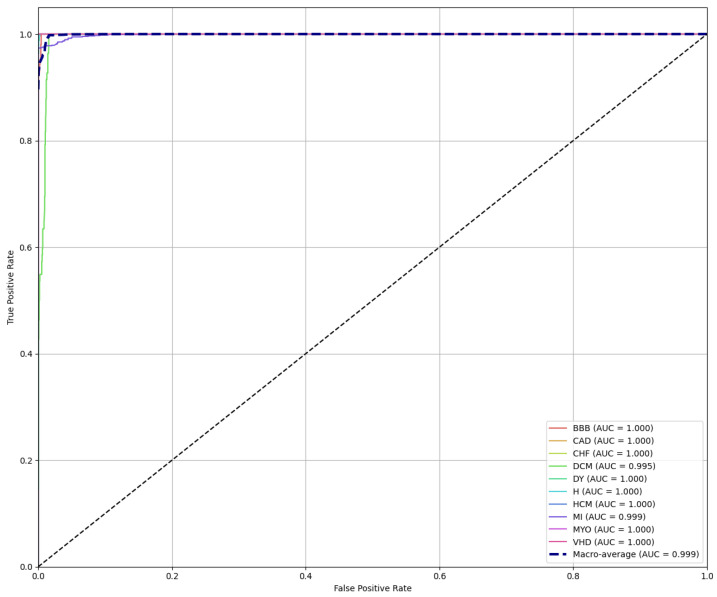
ROC curves for ten distinct classes.

Additionally, the Receiver Operating Characteristic (ROC) curve was constructed in Figure 17 to conduct a more detailed evaluation of the model’s performance. The ROC curve is a crucial indicator for assessing the performance of classification models. It underscores the variation in the true positive rate (TPR) as the false positive rate (FPR) increases. Notably, the figure clearly demonstrates the efficiency and effectiveness of the proposed model in distinguishing between classes.

The Area Under the Curve (AUC) values for both micro and macro averages are nearly 99.9%, attesting to the model’s capability and effectiveness in accurately classifying between the different classes.

**Figure 18:**
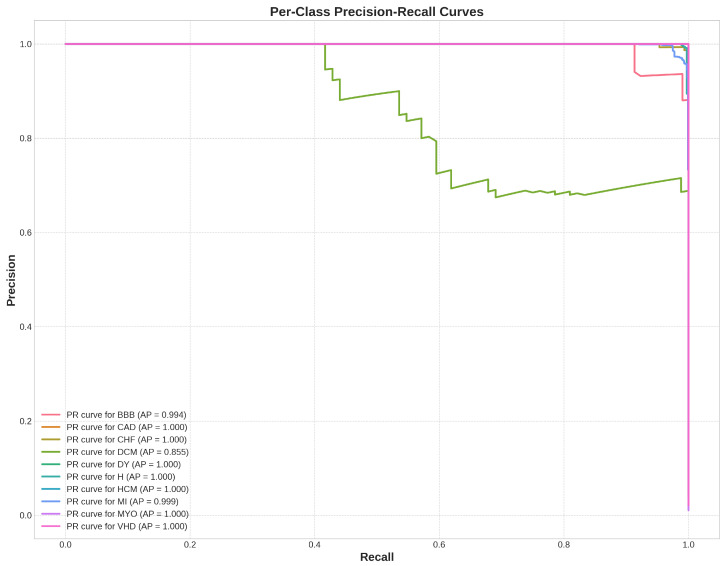
Precision-Recall curves for ten distinct classes.

To provide a more robust assessment of the model’s performance, particularly given the inherent class imbalance, per-class Precision–Recall (PR) curves were also generated, as shown in Figure 18. The Area Under the PR Curve, or Average Precision (AP), offers a robust summary of performance for each class.

Most classes achieved an AP near 1.0, indicating almost perfect classification. The overall Macro-Average Precision (mAP) of 0.9847 further confirms the model’s uniformly high performance across all categories. Notably, the PR curve for the DCM class shows a slightly lower AP of 0.932. This finding is consistent with our analysis of the confusion matrix, where DCM was identified as having a higher number of false positives, confirming that while the model is strong, it finds the DCM class marginally more challenging, which aligns with clinical expectations of overlapping ECG features.

### 4.2. Comparison with States-of-Arts Studies

Table 7 portrays a comparative performance evaluation of our model in comparison to baseline models. The comparison emphasizes the superior performance of our proposed approach in identifying cardiovascular abnormalities, while we have not performed direct statistical significance tests due to the varying evaluation protocols of the cited baselines, the consistent and notable margin of improvement in accuracy and F1-score suggests our model’s superior performance is robust.

In [26], a deep 1D-CNN was implemented for the classification of five cardiac condition types. Their method yielded respective values of 99.84% for accuracy, 99.52% for sensitivity, and 99.95% for specificity. However, they used a blind segmentation of 3-second duration, which impacted the computational efficiency and training time.

The authors in [28] introduced a classifier that utilizes a 16-layer CNN combined with LSTM models to automatically classify N, CHF, CAD, and MI classes using 2s ECG segments.

The performance of the model was evaluated using the ten-fold validation technique, which was employed to evaluate the model’s performance, yielding an accuracy of 98.5%. Nevertheless, deep architectures require a significant volume of data for effective training, making the process time-intensive and requiring significant computational capabilities.

In [25], the authors introduced an automated method for categorizing subjects as normal, CHF, MI, and CAD classes using 2-second ECG signals. Their research performed a performance comparison between a deep convolutional neural network (CNN) model with eight convolutional layers and a deep Gabor-CNN model. While the Gabor-CNN model requires less processing power than the deep convolutional neural network model, it still requires a large volume of samples to reach high performance. The Gabor-CNN and CNN models obtained accuracies of 98.74% and 99.55%, respectively.

The authors in [38] proposed a system for classifying eight types of cardiovascular diseases using ECG heartbeats of 0.64 s. Their method yielded an accuracy of 98.3%, precision of 98.9%, sensitivity of 99.4%, and an F1-score of 99.2%. In comparison, our approach outperforms their approach in terms of accuracy and precision while handling classification across ten cardiovascular classes.

Overall, the comparative analysis clearly demonstrates that the proposed method outperforms other approaches, showing high classification performance by achieving improved classification accuracy despite being trained with less data and shorter segments. In addition to its reliability and efficiency, the proposed framework shows strong potential for scalability. Since each ECG signal is processed independently, the feature extraction pipeline can be efficiently parallelized to handle large datasets. Furthermore, the framework is highly adaptable for classifying a wider range of cardiovascular diseases; extending the model to new CVD classes would only require adjusting the final classification layer and retraining the model, as the core architecture is class-agnostic. Finally, the use of a lightweight residual attention network supports computational scalability, making it suitable for deployment in environments with limited computational power.

Our proposed approach demonstrates that a lightweight network can achieve highly competitive performance when coupled with a sophisticated, domain-specific feature representation derived from CWT and SIFT. This methodology deliberately prioritizes the balance between classification accuracy and computational efficiency, which represents a practical trade-off critical for clinical deployment but often secondary in more resource-intensive, end-to-end models like Transformers. A direct comparison against leading end-to-end architectures is therefore an important next step.

From a clinical perspective, our framework’s lightweight design is a major advantage for real-world use. It’s efficient enough to serve as a rapid screening tool in point-of-care settings, such as primary care clinics or emergency rooms. Moreover, its low computational cost makes it an ideal fit for deployment on wearable devices or remote monitors. The model’s interpretability is enhanced by the spatial attention mechanism, which allows it to highlight the specific regions in the time-frequency domain that were most influential for its decision. This offers clinicians a visual map of the pathology, a form of explainability that is crucial for building trust and moving from a ’black box’ to a more transparent clinical decision support system.

Despite the promising results, it is important to contextualize the results of this study. Firstly, the validation strategy has key limitations related to the data and the splitting method. The framework was validated exclusively on the benchmark PhysioNet repository, and the cross-validation was not performed with a strict subject-wise split. Our cross-validation approach, while not strictly subject-wise, was chosen to ensure a direct and fair comparison with several key state-of-the-art benchmarks cited in our study, which utilize similar validation protocols.

Therefore, while our findings are comparable within the field’s common practices, we strongly emphasize that future work must re-evaluate this framework using a strict patient-level separation to establish a more conservative and realistic estimate of its clinical utility. Furthermore, future efforts must also focus on validating the model on larger, more diverse clinical datasets to confirm its broader generalizability.

Secondly, our model was trained on relatively clean, preprocessed ECG signals. Its robustness to common real-world issues such as motion artifacts and electrode misplacement has not yet been rigorously evaluated. Future work should assess the model’s performance on raw, less-processed ECG data to determine its true clinical viability.

Thirdly, to mitigate the heterogeneity from using multiple databases, we harmonized the data by resampling all signals to a uniform frequency. However, we acknowledge that a more rigorous ’leave-one-database-out’ validation is an important future step to confirm the model is truly dataset-agnostic.

Finally, while this study validates the model’s high accuracy, its performance in a real-time clinical workflow remains unassessed. Furthermore, a formal calibration analysis of the model’s probabilities has not been performed, nor have decision thresholds for clinical triage been explored. Future work will therefore also focus on evaluating the model in a simulated environment to benchmark its inference speed, resource consumption, and clinical utility.

## 5. Conclusions

In the past decades, researchers have extensively exploited the potential of the electrocardiogram in several applications that cover clinical fields. The electrocardiogram involves the analysis of the inherent discriminative characteristics present in the ECG signal by providing crucial insights into the time-varying bioelectric potentials produced by the heart’s electrical activity. In this study, we proposed a novel approach for classifying ten cardiovascular classes using ECG heartbeats of short durations. Our method utilizes a hybrid feature representation by combining the Continuous Wavelet Transform with Scale-Invariant Feature Transform (SIFT) keypoint density maps; it transforms 1D ECG segments into three-channel 2D images with rich feature properties. This synergy presents a distinctive contribution in capturing relevant and distinct features from the ECG signal, thereby enhancing the classification performance. Initially, the Continuous Wavelet Transform was employed to transform the preprocessed one-dimensional ECG signal into the time-frequency domain, generating two-channel scalogram images. Specifically, the first channel of the image is a scalogram image generated with the Morlet wavelet type, while the second channel is extracted using the Mexican wavelet type; this provides complementary CWT-based morphological information. Subsequently, the SIFT algorithm is implemented on the first channel, the Morlet-based scalogram, to detect keypoints that represent unique features in the time-frequency representation. These resulting three-channel images were then used as input to our proposed network. To address the challenge of imbalanced classes, we utilized the Synthetic Minority Over-sampling Technique alongside Edited Nearest Neighbors and the focal loss in the training process for better generalization performance. Our experimental results demonstrate remarkable classification performance using a five-fold cross-validation technique.

## Figures and Tables

**Table 1:** ECG data acquisition details from each corresponding database.

Database	ECG Condition	Number of Participants	Sampling Rate (Hz)	Channels
PTB diagnostic	N	52	1000	12-lead
	MYO	4		
	MI	148		
	DY	14		
	BBB	15		
	VHD	6		
PTB diagnostic	HCM	8	1000	12-lead
	DCM	7		
BIDMC Congestive Heart Failure	CHF	15	250	12-lead
INCART	CAD	32	257	Two-channel

**Table 2:** The detailed architecture of the proposed Light-ResAttNet model.

Layer (Type)	Output Shape	Parameters
Input Layer	(128, 128, 3)	0
Conv2D (5 × 5, stride 2)	(64, 64, 32)	2432
BatchNormalization, ReLU	(64, 64, 32)	128
Stage 1: Res-Block		
Pre-Activation Res-Block	(64, 64, 64)	57,728
Stage 2: Res-Block		
Pre-Activation Res-Block (stride 2)	(32, 32, 128)	229,632
Stage 3: Res-Block + Attention		
Pre-Activation Res-Block (stride 2)	(16, 16, 256)	918,016
Spatial Attention	(16, 16, 256)	99
Classification Head		
Global Average Pooling 2D	(256)	0
Dropout (0.5)	(256)	0
Dense (Softmax)	(10)	2570
Total Trainable Parameters		1,212,077

**Table 3:** Sample distribution per class before and after SMOTEENN balancing.

Class	Samples Before Balancing	Samples After Balancing
BBB	415	4614
CAD	680	4613
CHF	600	4614
DCM	334	4614
DY	334	4614
H	1426	4612
HCM	108	4614
MI	4614	4612
MYO	93	4614
VHD	185	4614
Total	8789	46,135

**Table 4:** Hyperparameter settings for the proposed framework.

Component	Parameter	Value	Justification
CWT	Mother Wavelet (Channel 1)	mexh (Mexican Hat)	Selected as it is highly effective for localizing sharp, transient events like the QRS complex.
	Mother Wavelet (Channel 2)	morl (Morlet)	Chosen to identify oscillatory patterns and harmonic components within the ECG signal.
	Scales	1 to 64	Chosen to capture a broad range of clinically relevant frequency components in ECG signals.
SIFT	Contrast Threshold	0.04	Standard value proposed in the original SIFT algorithm to ensure feature robustness.
	Edge Threshold	10	Standard value proposed in the original SIFT algorithm to eliminate unstable edge features.
SMOTE	Number of Neighbors (k)	5	A commonly used value as recommended in the original SMOTE paper [44].
ENN	Number of Neighbors (k)	3	A standard value for effective data cleaning, based on original nearest neighbor editing rules [44].
Focal Loss	Focusing Parameter (γ)	2.0	As proposed for optimal performance in the original paper [43]
	Alpha (α)	0.25	As proposed to balance class importance in the original paper [43]

**Table 5:** The overall performance comparison of the four ablation studies.

Experiment	Accuracy (%)	Precision (%)	Recall (%)	F1-Score (%)
Scalogram-Based Morlet Wavelet	96.5	96.2	95.6	96.5
Scalogram-Based Mexican Hat Wavelet	96.3	95.2	95.5	96.5
SIFT Keypoint Density Map	96.70	96.40	95.53	95.64
Hybrid Method	99.60	97.38	98.53	97.37

**Table 6:** Per-class performance metrics.

Class	Accuracy (%)	Precision (%)	Recall (%)	F1-Score (%)
BBB	99.54	92.73	98.08	95.33
CAD	99.95	100.00	99.40	99.70
CHF	99.86	100.00	100.00	100.00
DCM	98.53	70.75	91.46	79.89
DY	99.95	98.80	100.00	99.40
N	99.77	98.58	98.86	98.72
HCM	100.00	100.00	100.00	100.00
MI	98.44	99.46	97.46	98.45
MYO	100.00	100.00	100.00	100.00
VHD	100.00	100.00	97.87	98.92
**Average**	**99.60**	**97.38**	**98.53**	**97.37**

**Table 7:** Performance comparison of the proposed method with existing approaches.

Study Ref.	Class Distribution	Seg. Type	Length (s)	Model Architecture	Evaluation Results
[26]	MI = 148 BBB = 14 N = 52 VHD = 6 HCM = 7 DCM = 6	Blind	3 s	Standard CNN with min–max Normalization	Accuracy = 99.50% Precision = 97.33% Sensitivity = 99.30%
[38]	MI = 148 VHD = 6 N = 52 CHF = 15 BBB = 15 CAD = 7 C = 18	Heartbeat	0.64	Combination of Continuous Wavelet Transform, 2D Wavelet Transform, and Capsule Networks	Accuracy = 98.3% Precision = 98.9% Sensitivity = 99.4% F1-Score = 99.2%
[25]	MI = 148 N = 92 CHF = 15 CAD = 7	Blind	2 s	Gabor-Filtered Convolutional Neural Network	Accuracy = 98.74% Precision = 97.50% Sensitivity = 98.74%
[28]	MI = 148 N = 92 CAD = 7 CHF = 15	Blind	2 s	Hybrid CNN-LSTM (Sequential Deep Learning)	Accuracy = 98.51% Specificity = 97.89% Sensitivity = 99.30%
**Present Work**	MI = 148 VHD = 6 BBB = 15 N = 52 CAD = 7 HCM = 5 CHF = 15 MYO = 4 DCM = 13	Heartbeat	0.64	Fusion of CWT and SIFT features, and Light-ResAttNet 2D CNN	**Accuracy = 99.60%** **Precision = 97.38%** **Sensitivity = 99.53%** **F1-Score = 97.37%**

## Data Availability

The PTB Diagnostic ECG database at https://www.physionet.org/content/ptbdb/1.0.0/ (accessed on 26 July 2025), the BIDMC Congestive Heart Failure ECG signals database at https://www.physionet.org/content/chfdb/1.0.0/ (accessed on 26 July 2025), and the INCART database at https://physionet.org/content/incartdb/1.0.0/ (accessed on 26 July 2025). The source code that supports the findings of this study is available from the corresponding author upon request.

## Abbreviations

The following abbreviations are used in this manuscript:

ECG	Electrocardiogram
CVD	Cardiovascular Disease
CWT	Continuous Wavelet Transform
ResNet	Residual Neural Network
CNN	Convolutional Neural Network
SIFT	Scale-Invariant Feature Transform
SMOTE	Synthetic Minority Oversampling Technique
MI	Myocardial Infarction
N	Normal
VHD	Valvular Heart Disease
DY	Dysrhythmia
CAD	Coronary Artery Disease
CHF	Congestive Heart Failure
DCM	Dilated Cardiomyopathy
HCM	Hypertrophic Cardiomyopathy
MYO	Myocarditis
BBB	Bundle Branch Block
